# Failure to oxygenate during cardiopulmonary bypass; treatment options and intervention algorithm

**DOI:** 10.1051/ject/2024026

**Published:** 2024-12-20

**Authors:** Gregory S. Matte, William L. Regan, Sarah I. Gadille, Kevin R. Connor, Sharon L. Boyle, Francis E. Fynn-Thompson

**Affiliations:** 1 Department of Cardiac Surgery, Boston Children’s Hospital 300 Longwood Avenue Boston MA 02115 USA

**Keywords:** Cardiopulmonary bypass, Oxygenator failure, Oxygenator change-out, Cardiopulmonary bypass emergency

## Abstract

Membrane oxygenator failure remains a concern for perfusion teams. Successful outcomes for this low-frequency, high-risk intervention are predicated on having written institutional protocols for both the oxygenator change-out procedure as well as how often the procedure is practiced by staff perfusionists. A recent review of peer-reviewed journal articles, textbooks and online resources revealed a lack of a unified intervention algorithm for failure to oxygenate during cardiopulmonary bypass (CPB). While an oxygenator change-out procedure may still be considered the gold standard for a confirmed device failure, temporizing measures exist that, in select cases, can afford time to the clinical team and even obviate the need for an oxygenator change-out procedure. We now consider the venous piggyback technique sourcing blood from the venous limb of the circuit a first-line intervention to afford enhanced patient safety while the clinical team decides on required interventions when oxygenator failure presents during CPB.

“Failure is not an option.”

Attributed to NASA Flight Director Gene Kranz of the Apollo 13 Moon landing mission, April 1970.

## Overview

Membrane oxygenator failure during cardiopulmonary bypass (CPB) remains a concern for perfusion teams, even though most perfusionists have never had the occasion to perform an emergent oxygenator change-out [1]. The reported incidence of oxygenator failure is likely underreported since not all incidents end up in reporting databases and because there is not a standard definition for oxygenator failure [1, 2]. Soo et al. reported that there were 50, 101, and 133 reported cases of oxygenator failure in the United States in 2009, 2010, and 2011, respectively [3]. Da Broi et al. reported in 2006 that in the United States alone, one patient per month dies as the result of the oxygenator failure change-out procedure during extracorporeal membrane oxygenator support (ECMO) or CPB [2]. Willcox commented in his 2023 letter-to-the-editor regarding reviewing the literature for oxygenator failures that there were reported “…instances where change-out was considered but not done, sometimes with periods of marked hypoxaemia” [1]. These reports highlight how essential it is for perfusion programs to plan for an oxygenator failure emergency. Successful outcomes for this low-frequency, high-risk intervention are predicated on having written institutional protocols for managing primary oxygenator failure as well as how often the procedure is practiced by staff perfusionists [3, 4].

It is of principal concern to qualify that failure to oxygenate with the primary membrane oxygenator does not universally require an oxygenator change-out. First, the team must determine if the oxygenator itself is the root cause and not a host of other possibilities [3–6]. Oxygenator change-out procedures have been performed during CPB, with subsequent follow-up revealing an alternate cause for apparent oxygenator device failure [3]. Table 1 lists primary considerations for assessing the patient and circuit when oxygenator failure is suspected during CPB and is based on team experience and published guidance [5–7]. Basic confirmations of overall sweep flow and FiO_2_ are done but also verification that an appropriate ventilation-to-perfusion (V/Q) ratio is in use. The consideration of the V/Q ratio is central to avoiding condensation buildup within the microfiber bundles which will consequently result in a wetting-out effect [8–10]. Manufacturer-specific interventions to treat a wetted-out device must be followed. Verification of sweep flow must also ensure that a minimum gas flow, as recommended by the manufacturer, is maintained through the device. The anesthesiologist should also be consulted for depth of anesthesia and muscle relaxation.

**Table 1:** Example of a confirmation of oxygenator failure algorithm and preparation for an oxygenator change-out.

Work with a backup perfusionist on the following:
1. Assess arterial and venous lines for color difference. Confirm in-line gas values with lab specimen if possible.
2. Confirm sweep gas is on with the proper source (and blender settings if in use).
3. Confirm proper ventilation-to-perfusion (V/Q) ratio.
4. Confirm integrity of sweep gas system all the way to the oxygenator exhaust port (vaporizer cap sealed – may bypass vaporizer altogether/turn off vaporizer). Discern proper function by tactile and audible means (sweep gas line to oxygenator creates pressure when disconnected from oxygenator and temporarily blocked).
5. Confirm function of flow meter (if in use).
6. Confirm proper blood flow (appropriate pump arterial line/system pressure and appropriate patient arterial pressure, correct tubing size selected on arterial controller, flow probe value verified, closure of recirculation/prime lines).
7. Change to 100% oxygen if not already on that source (may change to stand-alone E-cylinder with flow meter to rule out issues with normal sweep gas system).
8. Consider whether the oxygenator may be “wetted out”. Use manufacturer recommended guidelines for treatment. Terumo FX-05: Increase sweep rate to sigh oxygenator; max sweep of 5 LPM for 10 s (do not repeat). Sorin D101: Increase sweep rate to sigh oxygenator; max V/Q of 4:1 for ≤10 min. Terumo FX15-30: Increase sweep rate to sigh oxygenator; max sweep of 15 LPM for 10 s (do not repeat). Terumo FX-25: Increase sweep rate to sigh oxygenator; max sweep of 20 LPM for 10 s (do not repeat).
9. Consult with the anesthesiologist to confirm proper muscle relaxants/anesthesia are in use (check reported VO_2_ value). Consider malignant hyperthermia if the CO_2_ is significantly elevated with a low SvO_2_ (if an isoflurane source is in the sweep gas system).
10. Request second perfusionist to clear-prime a replacement oxygenator in the pump room with quick-connect tubing and connectors attached to replacement device.
11. Inform surgeon of findings and discuss action plan (define lowest acceptable PaO_2_ before change-out). If change-out required, clarify if it will be with a relatively warm and ventilated patient that is ejecting versus hypothermic with circulatory arrest.

Once primary oxygenator failure has been verified, the clinical team must decide on an intervention, or series of interventions, with timing dependent on the PaO_2_, SaO_2_, patient temperature, surgical progress, and native circulatory status. Table 2 lists recommended steps for a traditional oxygenator change-out procedure, which requires an interruption of cardiopulmonary support [5, 7]. This was the internal guidance we had for an oxygenator change-out before our most recent case of oxygenator failure.

**Table 2:** Traditional oxygenator changeout procedure requiring temporary interruption of extracorporeal support. These steps occur after multidisciplinary discussion that determines the PaO_2_ change-out threshold, the patient temperature for the procedure, and timing. The perfusion team communicates progress to the care team during the procedure.

Oxygenator change-out procedure
1. Prepare sterile cut locations: scissors, betadine/alcohol, towels, and flush solution for connections.
2. Primary perfusionist to decide if oxygenator-only change-out will be performed versus an oxygenator-reservoir change-out.
3. Confirm sufficient venous reservoir volume for procedure.
4. Come off bypass and clamp arterial and venous lines (drain patient vs. fill up per status of native cardiopulmonary function).
5. Replace oxygenator (± reservoir) with new clear-primed device using precut/clamped segments of tubing with connectors already attached. Use flush solution for connections as needed.
6. Move sweep gas line to new oxygenator.
7. Flow through recirculation limb and verify circuit is deaired.
8. Perform re-establishing bypass checklist.
9. Initiate CPB.
10. Perform secondary checklist once back on CPB.
Reestablishing bypass checklist
1. Tubing connections correct and tight.
2. Circuit deaired.
3. Sweep gas on and line reconnected.
4. Tubing clamps off boot.
5. Extra clamp(s) removed from arterial line.
6. Recirculation line and purge line clamped.
7. Pressure dome and manifold line reconnected and opened.
8. Communicate circuit status with surgeon.
Secondary back-on checklist
1. Verify blood color and Terumo CDI values.
2. Change over water lines.
3. Reconnect temperature probe(s).
4. Tie band connections.
5. Connect WAGD line.

*WAGD = waste anesthesia gas disposal.

The most recent case caused us to reconsider all options for such an emergency. This report summarizes intervention options for oxygenator-reservoir device failure along with our team’s updated oxygenator failure intervention algorithm. It also includes our bypass circuit framework that allows a quick and efficient intervention to provide venous piggyback oxygenation, which may serve as a temporizing measure with or without an eventual oxygenator change-out. A programmatic overview of CPB oxygenator issues and a recent case are also discussed. An Institutional Review Board (IRB) waiver was granted for this manuscript (IRB-P00049148).

## Intervention options for oxygenator-reservoir failure during CPB

Perfusionists have options to consider when an oxygenator shows signs of failure during cardiopulmonary bypass. Groom et al. reported on the parallel replacement of an oxygenator not transferring oxygen (PRONTO) technique in 2002 [11]. This technique is seemingly ideal because a definitive replacement may be performed without interrupting surgical progress and without circulatory arrest. Over twenty years have passed since their article was published, yet based on anecdotal evidence, the technique has not garnered widespread adoption. Not all perfusion programs are even aware of the option [3]. Programs familiar with the PRONTO technique may be hesitant to adopt it considering the custom tubing pack changes required and additional Y-connections pre and post- the primary oxygenator for all setups. Additional connectors and flow paths on 100% of pump circuits introduce areas of risk that must be considered against the rare occurrence of true oxygenator failure requiring replacement during CPB. Furthermore, unlike in 2002, when the PRONTO technique article was published, the post-oxygenator connection is now commonly post-arterial line filter (ALF) with the advent of integrated ALFs. That adds another concern in that there is no longer a final filter between the intervention site in the circuit and the patient. Grist [1] and Willcox [12] have both published endorsements for the PRONTO technique in the past few years for good reason; teams must be prepared to deal with oxygenator failure for optimal patient outcomes and patient safety. While an oxygenator change-out procedure may still be the gold standard, alternative temporizing measures exist that, in select cases, can afford time to the clinical team and even obviate the need for an oxygenator change-out. These measures are of critical consideration for programs not electing to include connections for a PRONTO setup.

**Figure 1:**
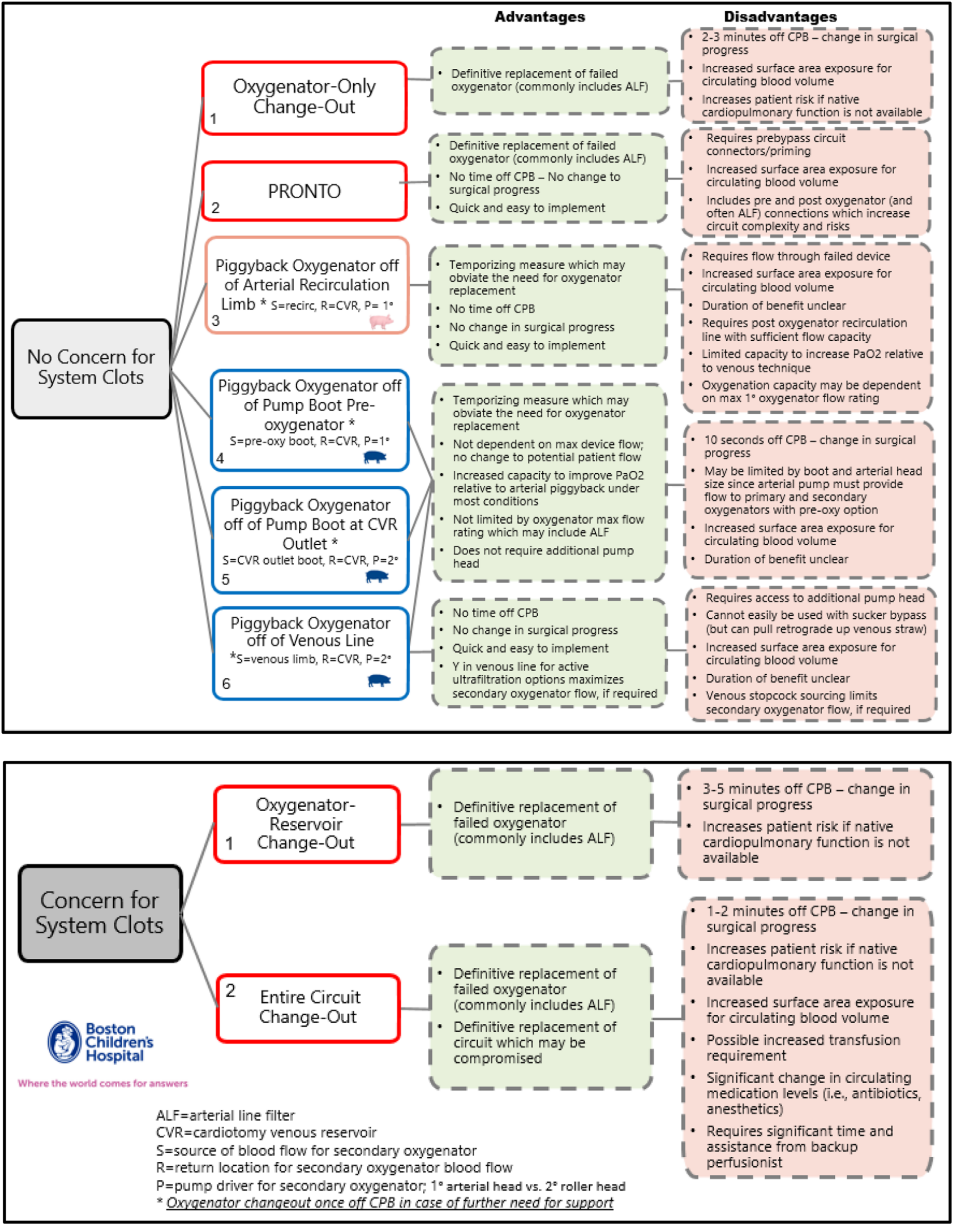
Overall options to treat confirmed oxygenator failure with and without concern for system clots during cardiopulmonary bypass.

Figure 1 shows an internally developed document summarizing our team’s experience and thought-framework for options to treat confirmed oxygenator failure. Key advantages and disadvantages of each method are included. A primary consideration that must be taken into account during the assessment of declining oxygenator function is whether there is a concern for clots in the extracorporeal circuit. We have identified six options to treat oxygenator failure when there are no concerns for system blood clots and two options when concerns for system clots are present.

**Figure 2:**
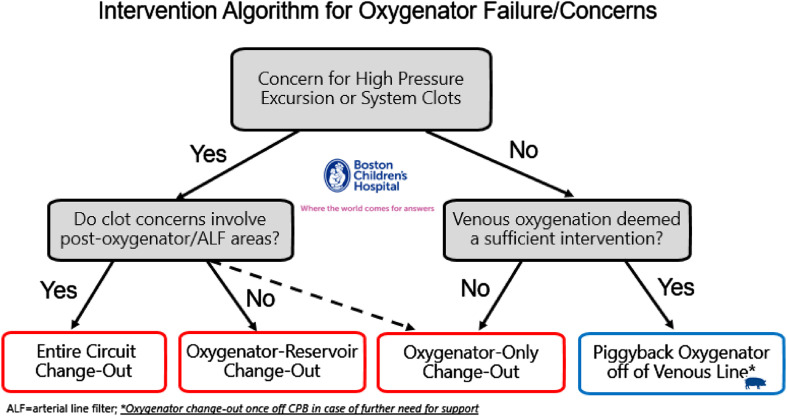
Our institution’s updated intervention algorithm for confirmed oxygenator issues during cardiopulmonary bypass.

### Oxygenator failure without concern for system blood clots

Isolated oxygenator failure can present at any time during CPB, although most often, it is a progressive change over time that affords some notice to the team [5]. An oxygenator-only change-out was long considered the gold standard intervention as it offered a definitive replacement of the failed device. Most books and review articles mention the technique without alternatives [5, 7, 13]. The PRONTO technique was a novel and important development since it offered a method for definitive replacement without the need to come off of CPB. However, there have been concerns with implementing universal circuit changes, particularly post-ALF, as previously described. Clinicians have sought other options that, while not offering definitive replacement of a failed device, offer time to the clinical team. Impressively, these other techniques, which piggyback oxygenation with a secondary device inserted into one of several locations in the pump circuit, may obviate the need to replace the oxygenator during CPB.

The first of these alternative techniques may be termed an arterial piggyback. Here, the patient does not need to come off bypass as a secondary oxygenator is inserted into an arterial recirculation limb of sufficient caliber to provide adequate secondary oxygenator flow with a return to the cardiotomy venous reservoir (CVR). This effectively pre-oxygenates the blood before passage through the failing primary device. The disadvantages of this technique are that the recirculation limb may be insufficient to provide secondary oxygenator flow and that the arterial head must provide flow to both devices. Overall output may be limited at the upper end of potential flow for a given boot in an arterial raceway since the flow to the primary and secondary oxygenators are additive. Additionally, the maximum rated flow for the primary oxygenator, which is still in line, may limit the capacity for secondary oxygenator flow since the secondary oxygenator flow is a shunt off of the post-primary-oxygenator recirculation limb. The maximum recommended flow from the manufacturer may be linked to the efficiency of an integrated arterial line filter, so clinicians must heed caution and not exceed that limit.

There are three other piggyback oxygenation techniques, and they all source blood from the venous side of the circuit. The first requires approximately 10 s off CPB to cut a Y-connector with tubing attached to the pre-oxygenator boot line. This allows the team to connect a secondary oxygenator into the circuit with the primary arterial pump providing flow to both oxygenators. The secondary oxygenator blood flow then returns to the CVR. The second venous piggyback option also requires a brief period off CPB to cut a similar connector with tubing into the CVR-outlet boot line. This addresses the limitations of the arterial boot tubing size and roller head size since flow to the piggyback device is provided with a separate pump head. Here, the secondary roller head pump is configured to draw venous blood from the CVR, pump it through the secondary oxygenator, and then back to the CVR. The third and likely most attractive option is to provide piggyback oxygenation to the venous blood, sourcing it from the venous line and returning it to the CVR. This method is dependent on the available venous line connections for flow capacity. A Y-connection would provide the best flow capacity while a traditional venous limb stopcock may work but could be limiting depending on the level of device failure and required flow to the secondary oxygenator to meet overall oxygen transfer needs.

### Oxygenator-reservoir failure with concurrent concern for system blood clots

Clotting within the circuit during CPB is a significant concern since emboli can cause neurologic deficits and stroke [14]. Both intervention options listed in Figure 1 include definitive device change-outs with either the oxygenator reservoir being replaced or the entire CPB circuit. These interventions require relative expediency in action and a period of time off support, which may place the patient at risk if native cardiopulmonary function is not available. An entire circuit change-out exposes the patient to significantly more circuit surface area that may affect the systemic inflammatory response. It may also increase transfusion requirements depending on starting hematocrit, patient circulating blood volume, circuit size, and the ability to recover red cell mass from the original circuit. Changes in circulating medication levels also need to be considered, with redosing for antibiotics and anesthetics likely warranted.

Ultimately, intervention options for confirmed oxygenator-reservoir issues will vary depending on pre-bypass circuit configurations, team preference, and institutional experience.

## Programmatic review of oxygenator device issues

Boston Children’s Hospital has an internal non-routine event reporting system where all cases of issues during cardiopulmonary bypass and perfusion services are reported [15]. These events are discussed at a quarterly multidisciplinary conference led by the perfusion team as part of a quality assurance and improvement initiative. Over the past 15 years, we have documented seven instances of suspected oxygenator failure or other oxygenator performance-related issues. Two cases required no circuit intervention during or after bypass as the PaO_2_ was deemed sufficient at >100 mmHg on 100% oxygen and there was a low chance of resuming CPB. In two cases, the perfusion team changed the oxygenator only after normally weaned from bypass, in case an additional period of CPB was required. One case was treated with an oxygenator-only change-out during a surgically-planned period of circulatory arrest at 18 °C. The sixth incident involved concern for visible clots in the post-oxygenator/integrated ALF part of the bypass circuit that progressively developed during CPB. The care team elected to perform an entire circuit change-out for that case (later follow-up revealed the area of concern to be aggregated platelets and not fibrin). The seventh and most recent case in our experience showed oxygenator failure during the cross-clamp period at approximately 6.5 hours on CPB. The perfusionists successfully employed an arterial piggyback method to support the primary oxygenator for that case. There were no instances of emergent, on-bypass, oxygenator change-out in the past 15 years at our institution, which included over 13,000 cardiopulmonary bypass cases.

### Recent experience

The seventh reported case of oxygenator failure in our experience, mentioned above, included a 452-minute bypass run on a patient with compromised pulmonary function. That 7 kg patient circuit had a 3/16″ arterial and post-oxygenator recirculation limb, as well as a ¼″ venous limb on a Stockert S5 heart-lung machine (LivaNova PLC, London, UK). It was understood preoperatively that there was a high likelihood that the patient would be transferred to extracorporeal membrane oxygenation (ECMO) post-cardiotomy before transfer to the intensive care unit (ICU). Two perfusionists were available to support the primary perfusionist while assessing the slowly declining PaO_2_. The surgeon and anesthesiologist were informed of the developing issue and a second Terumo CAPIOX FX-05 oxygenator (Terumo Cardiovascular Systems, Elkton, MD) was primed in the pump room with heparinized (3 IU/mL) Plasma-Lyte A 7.4 (Baxter Healthcare Corporation, Deerfield, IL). The oxygenator bundle was prepared with 3/16″ inlet and outlet connectors to facilitate a time-efficient oxygenator change-out, as regularly practiced by the perfusion team. The care team verified that the oxygenator was indeed failing per written institutional guidance that has the team rule out numerous potential contributing factors (Table 1) [5]. Over the course of 37 min (CPB time 397–434 min), the PaO_2_ on 100% oxygen declined from 250 mmHg to 98 mmHg. This occurred despite serial increases in sweep flow rate, an oxygen source change to a standalone tank, and a Terumo FX-05-specific oxygenator “wet-out” procedure of increasing the sweep rate to 5 LPM for only 10 s (without repeating per the instructions for use) [16]. The team decided that since the patient would separate from the bypass in another 15–20 min and likely be transitioned to ECMO, an arterial piggyback would be sufficient to support oxygenation. The primed secondary oxygenator was inserted into the standard 3/16″ arterial recirculation line that is included on all of our institution’s neonatal and infant circuits. The arterial pump flow was set at the primary device’s maximum recommended flow of 1500 ml/min with partial clamping of the post-primary oxygenator recirculation line that led to the secondary oxygenator. This resulted in a patient flow of 860 mL/min (cardiac index of 2.3 mL/min/m^2^) and secondary oxygenator flow of 640 mL/min, and we thought this was a reasonable balance between systemic flow and secondary oxygenator flow. An arterial flow probe (Transonic Systems, Inc., Ithaca, NY) was used to guide effective patient flow. The in-line Terumo CDI monitor (Terumo Cardiovascular Systems, Elkton, MD) indicated a rising PaO_2_, which settled at >200 mmHg with 100% oxygen sweep gas running to both the primary and secondary oxygenator. It was of principal consideration that the clinical care team timed the arterial piggyback intervention to prevent hypoxemia and a subsequent decrease in venous oxygen saturation (SvO_2_), cerebral NIRS values and indexed oxygen delivery. Of note, the nadir SvO_2_, cerebral NIRS (left/right) and indexed oxygen delivery values during the progressive oxygenator failure period were 70%, 85%/85%, and 409 mL/min/m^2^, respectively. The patient was separated from CPB and was immediately transitioned to venoarterial ECMO as native pulmonary function was deemed insufficient.

## Discussion

Our perfusion team has written policies and procedures that are regularly reviewed and updated. Emergency drills are practiced and discussed in groups of 2–6 perfusionists at least every four months, with participation documented in departmental files. Additionally, annual multidisciplinary team training drills incorporate CPB emergencies, including oxygenator failure. This culture of actively training in different settings helps ensure optimal outcomes when this low-frequency, high-risk emergency presents during clinical practice.

A debrief was held after the recent oxygenator failure incident. The arterial piggyback technique worked during the most recent case of failure but would have limitations for other cases, given patient flow requirements relative to the device’s maximal flow. The arterial piggyback method creates a dependent and inverse relationship between patient flow and PaO_2_. Increasing the systemic flow decreases secondary oxygenator flow, and thus the achievable PaO_2_, if working at the limit of the manufacturer’s rated flow. This may not be of clinical relevance if the patient’s blood flow is on the low end of an oxygenator device’s overall flow rating. This dependency is of clinical concern if the patient’s blood flow requirement during support is near a device’s overall maximal blood flow rating since there may be insufficient capacity for secondary oxygenator flow to increase the PaO_2_. Again, this concern would be important if working at the limit of an oxygenator’s manufacturer-defined maximum flow rating, which may be linked to the integrated ALF flow efficiency.

**Figure 3:**
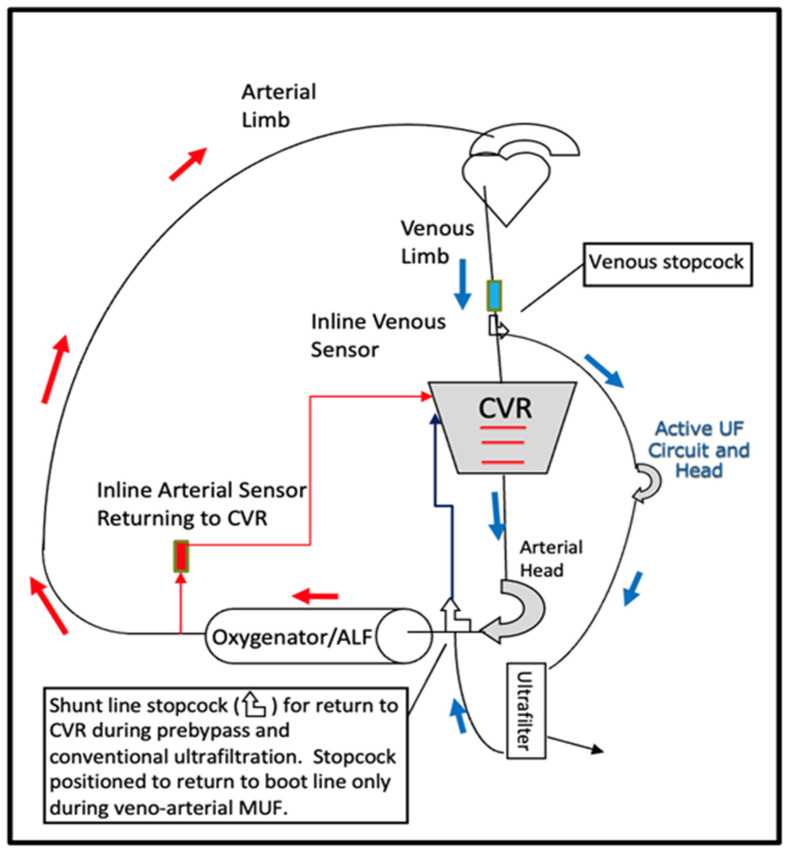
Schematic of CPB circuit with standard active ultrafiltration head incorporated. Blood can be sourced from the venous line for pre-bypass, conventional, and modified venoarterial ultrafiltration. This option exists on all circuit sizes.

The dependent relationship between the primary and secondary oxygenator during an arterial piggyback had the perfusion team consider a venous piggyback method (venous-assisted oxygenation) as a better alternative to the arterial piggyback method for future cases. We believe that the venous piggyback technique of sourcing blood from the venous limb of the circuit is preferable to the other piggyback techniques. Here, blood is sourced from the venous limb, run through a secondary oxygenator, and then back to the primary CVR. Preoxygenating the venous blood in this way occurs without dependency on the overall systemic blood flow, as with the arterial piggyback technique and, to some degree, the venous piggyback technique which sources blood from the pre-oxygenator boot line. Oxygenation support and pump flow to the patient are independent considerations. Of course, the heart-lung machine needs a secondary roller head pump dedicated to this task, and blood-sourcing capacity from the venous line would need to be sufficient to support the desired flow rate to the secondary oxygenator. A stopcock on the venous limb of the circuit is nearly universal. A pump head pulling off the venous line may not be common in perfusion practices, but ours allows for it in all cases. We can utilize active ultrafiltration with a dedicated mini-pump head on all circuit sizes, sourcing blood from the venous line through a high-flow stopcock, running it through a hemoconcentrator, and then returning it to the CVR, as shown in Figure 3.

**Figure 4:**
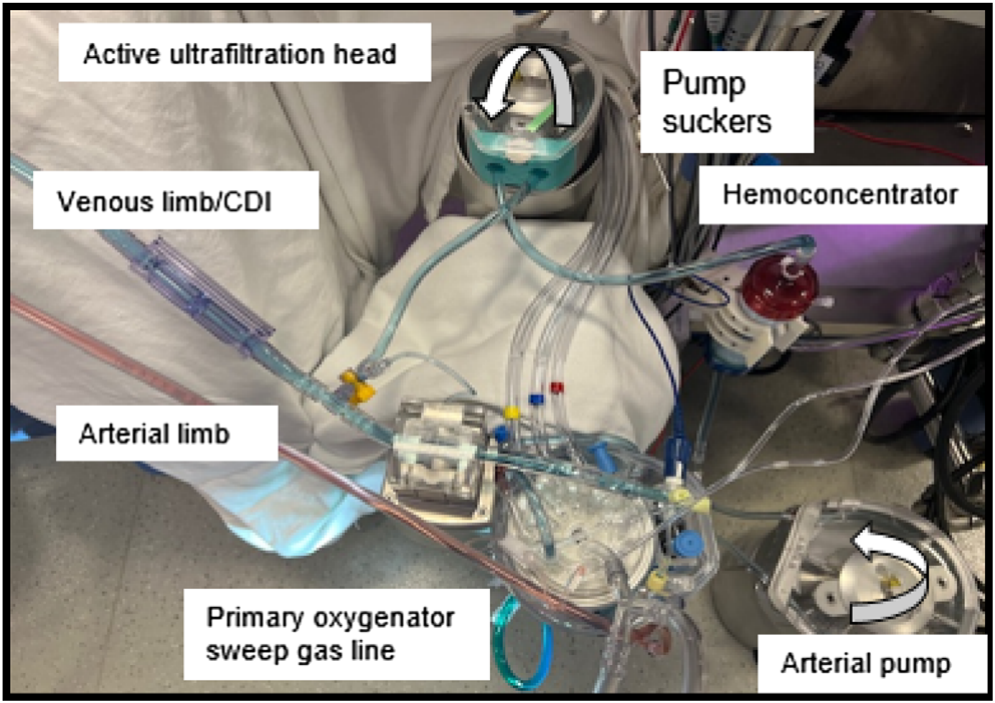
Picture of a Terumo CAPIOX FX-05 oxygenator with a neonatal/infant 3/16″ arterial limb by ¼″ venous limb circuit, with a stopcock on the lured venous line connection for the active ultrafiltration circuit. Note that the post-hemoconcentrator line returns to the cardiotomy reservoir for pre-bypass and conventional ultrafiltration (not shown in the image).

Our practice also utilizes pre-bypass, conventional, and modified venoarterial ultrafiltration on most neonates, infants, and children under 25 kg using the same mini pump head [17–19]. A schematic of the circuit setup is shown in Figure 3, with a bird’s-eye view picture of our standard circuit arrangement depicted in Figure 4. Venous-assisted oxygenation using a secondary oxygenator sourcing blood through the venous limb stopcock is thus easily implemented in our practice. While the venous piggyback technique had not been part of our policy and procedure manual prior to the most recent incident, it has since been added to our intervention algorithm, per Figure 2.

Upon reviewing the literature, we found that Boettcher et al., reported in 2017 that they used a similar technique as a temporizing measure during oxygenator failure in pediatric patients [20]. That team experienced five oxygenator failure incidents over a four-year span. A venous piggyback technique sourcing blood from the venous limb allowed them to avoid an oxygenator change-out and interruption of surgical progress in all cases. Subsequently, they included an addendum stating that two oxygenator failures in adult patients were successfully treated with the inclusion of a *pediatric* oxygenator cut into their standard ¼″ arteriovenous bridge line.

**Figure 5:**
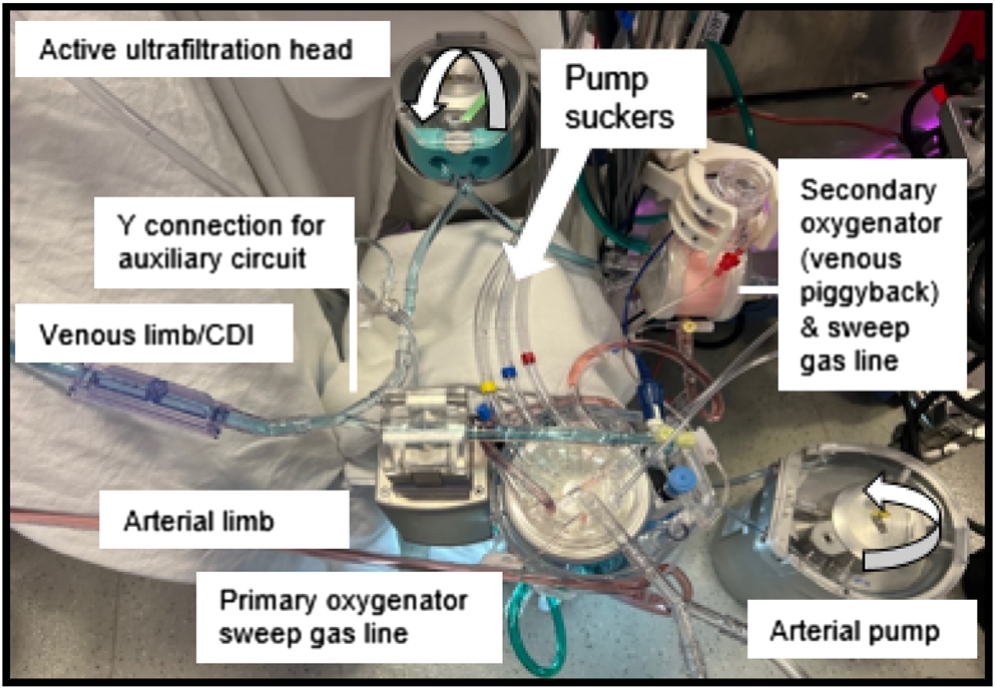
Picture of a Terumo CAPIOX FX-05 oxygenator with a neonatal/infant 3/16” arterial limb by ¼” venous limb circuit, modified with venous limb Y to allow for maximal secondary oxygenator flow (instead of standard venous line stopcock). For instructional purposes, the image depicts a secondary oxygenator replacing the hemoconcentrator for venous-assisted oxygenation (venous piggyback). Secondary oxygenator flow returns to top of CVR.

Secondary oxygenator flow capacity for a venous piggyback technique was an important qualification our team had been considering. Specifically, how do we ensure sufficient flow to the secondary device since flow through a stopcock can be limiting? We do not have an arteriovenous bridge in our selection of circuits like the Boettcher group. In our practice, one solution would be to replace the active ultrafiltration-sourcing stopcock (pre-bypass, conventional, and modified ultrafiltration techniques) with a Y-connection as a circuit standard as shown in Figure 5. We believe this simple circuit modification would allow for ample venous assisted oxygenation and afford time and safety to the clinical team to decide on interventions. This method for venous piggyback oxygenation may even obviate the need for an oxygenator change-out if PaO_2_ support was the original concern.

Figure 5 also depicts the replacement of the hemoconcentrator with a secondary oxygenator for illustrative purposes. Certainly, if a team builds their circuits with sufficient auxiliary venous flow access (i.e., a venous Y-connector for active ultrafiltration techniques instead of a stopcock), removing the hemoconcentrator would not be necessary. In fact, our standard circuit with active ultrafiltration includes an option for traditional passive ultrafiltration during CPB, which would not interfere with the flow path for a venous piggyback technique if the need arose. Passive ultrafiltration could continue as needed, with the active ultrafiltration head providing secondary oxygenator flow without the need to separate from bypass.

Considering our recent experience and discussion at our multidisciplinary clinical practice meeting, we have updated our oxygenator failure intervention algorithm, as shown in Figure 4. An oxygenator change-out procedure may be a rare occurrence, but when it does occur during CPB, teams must be exceptionally prepared. A cautionary tale by Moore et al., in 2002 made this point well in their report, highlighting the need to perform two oxygenator change-out procedures during the same bypass case [21]! We believe perfusion teams should revisit their oxygenator failure intervention options since temporizing measures exist, which may prove useful during patient care. We believe that our most recent experience with oxygenator failure included a well-timed and appropriate intervention that prevented both patient hypoxemia and circulatory arrest, as evidenced by the nadir SvO_2_, bilateral cerebral NIRS, and indexed oxygen delivery values. Gene Kranz, NASA Flight Director for the Apollo 13 Moon landing mission, is credited with the statement that, “Failure is not an option.” For cardiac operating room teams, failure of an oxygenator bundle will happen, but failure to intervene appropriately must not occur.

## Conclusion

Oxygenator failure is a high-risk, low-frequency event that perfusion teams must be prepared to handle. An oxygenator change-out procedure has long been considered the standard intervention, with or without cessation of cardiopulmonary support, depending on surgical progress and a team’s standard bypass circuit configuration (i.e., the inclusion of a PRONTO option). We have modified our clinical practice to include an intervention algorithm for confirmed oxygenator device failure. We now consider the venous piggyback technique, sourcing blood from the venous limb of the circuit, a first-line intervention to afford enhanced patient safety while the clinical team decides on required interventions when oxygenator failure presents during CPB.

## Data Availability

All data pertinent to this manuscript is included in the text.
